# A brief history of acute stroke care

**DOI:** 10.18632/aging.101542

**Published:** 2018-08-29

**Authors:** Rahul Damani

**Affiliations:** 1 Department of Neurology, Baylor College of Medicine, Houston, TX 77030, USA

**Keywords:** acute ischemic stroke, history, tPA, thrombectomy, thrombolysis

Stroke is leading cause of disability and fifth leading cause of mortality in the US. Approximately 800,000 stroke occurs in the US each year which translates roughly in one stroke every 40 seconds [1]. Not only stroke is an important healthcare burden but with estimated direct and indirect cost of stroke care over $100 billion a year it is also an economic one.

Despite the magnitude of problem there was therapeutic nihilism for patients with acute ischemic stroke for centuries. However, in 1995 a landmark NINDS tissue-type plasminogen activator (tPA) trial [2] created paradigm shift in management of acute ischemic stroke. This randomized controlled trial showed tPA improved functional outcome at 90 days in acute stroke with Number Needed to Treat (NNT) of 7.7. Since then there have been multiple randomized controlled trial including ECASS III which showed efficacy of tPA up to 4.5 hours from symptom onset albeit at higher NNT of 13.7. A 2014 individual patient level meta-analysis of all tPA randomized trials showed efficacy of tPA in 0-4.5 hour time window, irrespective of age and severity of stroke but with strong relationship with treatment time [3]. However, given small therapeutic window for treatment only a fraction of eligible patients with acute ischemic stroke receive tPA. There is room for individual (hospital) and system level (regional, county, state and national) improvements to maximize the benefits of tPA, by giving it to more eligible patients and by giving it faster. Such improvements at individual hospital level can and has been achieved by creating a protocol based approach of early identification and treatment of acute stroke patients, multidisciplinary stroke teams, education and awareness among EMS & emergency nurses, mobile stroke units and establishing telemedicine/tele-stroke services for small rural hospital, however similar improvement at system level has been elusive. Need of hour is to develop simple, novel, effective, cost neutral, easy to implement intervention which would not only increase systemic thrombolysis rate for eligible stroke patients but also reduce their door to needle (DTN) times on a larger scale. One of such example comes out of Southeast Texas study [4]. This regional intervention study was designed to investigate effect of un-blinding data on tPA administration and sharing data with CEO of 26 participating hospital of Southeast Texas Regional Advisory Council (SETRAC). Study showed that simple intervention of bringing transparency and un-blinding data across region encouraged CEO to implement strategies to improve stroke care at hospital level which resulted in 21% increase in tPA administration rate across region with 38% increase in tPA administration with DTN time of ≤ 60 minutes [4]. Intervention also resulted in average lifetime cost saving of $3.6 million.

The history of endovascular treatment with intra-arterial tPA and later mechanical thrombectomy (MT) is even younger than systemic thrombolysis. The impetus to develop effective endovascular therapy was based desire to expand 4.5 hours window of acute stroke treatment. However, road to it was a rocky one. One of the earlier intra-arterial tPA trial (PROACT II) ended in equipoise this was followed by multiple single arm device trials and three failed randomized control trial (SYNTHESIS, MR RESCUE, IMS III). This period of failed trials over many years led researchers to introspect and identify potential improvements that could be made with use of different technique devices and better patient selection using advanced imaging technique. The watershed movement came in early 2015 when a randomized controlled trial out of Netherlands (MR CLEAN) [5] showed positive outcome from MT in acute ischemic stroke patients with large vessel occlusion (LVO) performed within 6 hours of symptoms onset, with impressive NNT of 7.4 to reduce functional disability at 90 days. Results of MR CLEAN prompted multiple other ongoing RCT (EXTEND IA, ESCAPE, SWIFT PRIME, REVASCAT & THRACE) to perform interim analysis, all of which showed positive results. A recently formed HERMES collaboration performed patient-level meta-analysis [6] of above 5 randomized controlled trials showed NNT of 5.1 for improved functional outcome at 90 days with mechanical thrombectomy for patients with LVO stroke in anterior circulation if performed within 6 hours. MR CLEAN was dubbed as “step in right direction” and rightly so. The reason for such stark difference in results from earlier RCT are multifactorial. The newer RCT required mandatory advanced vascular imaging to identify patients with proximal occlusion, use of advanced retrievable stent and rapid door-to-recanalization times. Another breakthrough came in 2018 with DAWN [7] and DEFUSE 3 [8] trials. DAWN trial showed efficacy of MT in patients with anterior LVO presented between 6 and 24 hours after stroke onset with documented mismatch between the severity of the clinical deficit and the infarct volume (penumbral tissue). This resulted in impressive NNT of 3 for improved functional outcome at 90 days. Similarly DEFUSE 3 trial showed efficacy of MT in patients with anterior LVO presented between 6 and 16 hours after stroke onset with documented mismatch (penumbral tissue) with NNT of 3.5 for improved functional outcome at 90 days. The results of DAWN and DEFUSE 3 trials has essentially replaced “timed window” with “tissue perfusion window” in proportion of patients with acute stroke.

Just under a quarter of century we have come a long way from therapeutic nihilism to one with multiple proven options. Reducing time of onset of stroke to treatment and providing all therapeutic options to eligible patients remains cornerstone and will results in best outcomes. While statistics of stroke outcomes have looked bleak till now, future of stroke care looks promising.

## References

[1] Ovbiagele B, Nguyen-Huynh MN. Neurotherapeutics. 2011; 8:319–29. 10.1007/s13311-011-0053-1 21691873 PMC3250269

[2] National Institute of Neurological Disorders and Stroke rt-PA Stroke Study Group. N Engl J Med. 1995; 333:1581–87. 10.1056/NEJM199512143332401 7477192

[3] Emberson J, et al. Lancet. 2014; 384:1929–35. 10.1016/S0140-6736(14)60584-5 25106063 PMC4441266

[4] Damani RH, et al. Stroke. 2018 STROKEAHA.118.021109. 10.1161/STROKEAHA.118.021109 29991653

[5] Berkhemer OA, et al. N Engl J Med. 2015; 372:11–20. 10.1056/NEJMoa1411587 25517348

[6] Goyal M, et al. Lancet. 2016; 387:1723–31. 10.1016/S0140-6736(16)00163-X 26898852

[7] Nogueira RG, et al. N Engl J Med. 2018; 378:11–21. 10.1056/NEJMoa1706442 29129157

[8] Albers GW, et al. N Engl J Med. 2018; 378:708–18. 10.1056/NEJMoa1713973 29364767 PMC6590673

